# Providing low-dimensional feedback of a high-dimensional movement allows for improved performance of a skilled walking task

**DOI:** 10.1038/s41598-019-56319-9

**Published:** 2019-12-24

**Authors:** Kevin A. Day, Amy J. Bastian

**Affiliations:** 1 Center for Movement Studies, Kennedy Krieger Institute, Baltimore, MD 21205 USA; 2 Department of Biomedical Engineering, The Johns Hopkins University School of Medicine, Baltimore, MD 21205 USA; 3 Department of Neuroscience, The Johns Hopkins University School of Medicine, Baltimore, MD 21205 USA

**Keywords:** Motor control, Human behaviour, Biomedical engineering

## Abstract

Learning a skilled movement often requires changing multiple dimensions of movement in a coordinated manner. Serial training is one common approach to learning a new movement pattern, where each feature is learned in isolation from the others. Once one feature is learned, we move on to the next. However, when learning a complex movement pattern, serial training is not only laborious but can also be ineffective. Often, movement features are linked such that they cannot simply be added together as we progress through training. Thus, the ability to learn multiple features in parallel could make training faster and more effective. When using visual feedback as the tool for changing movement, however, such parallel training may increase the attentional load of training and impair performance. Here, we developed a novel visual feedback system that uses principal component analysis to weight four features of movement to create a simple one-dimensional ‘summary’ of performance. We used this feedback to teach healthy, young participants a modified walking pattern and compared their performance to those who received four concurrent streams of visual information to learn the same goal walking pattern. We demonstrated that those who used the principal component-based visual feedback improved their performance faster and to a greater extent compared to those who received concurrent feedback of all features. These results suggest that our novel principal component-based visual feedback provides a method for altering multiple features of movement toward a prescribed goal in an intuitive, low-dimensional manner.

## Introduction

Complex movements can be broken down into their constituent parts that vary over position and time in a coordinated manner. Whether it is the rotation of the shoulders, hips, and torso at specific times during a golf swing or the flexion/extension of the knee, shoulder, elbow, and wrist joints needed to execute a free throw shot in basketball, movements on a whole-body scale require coordination on the individual joint scale. A challenge when trying to alter these types of multi-jointed movements is that interactions between individual joints prevent us from manipulating one joint in isolation without impacting the others. Indeed, the principle ‘the whole is greater than the sum of its parts’ applies to complex movements, as a multi-jointed movement is not simply derived from the summation of the motor commands necessary to control individual joints^1–3^. When manipulating a walking pattern specifically, these principles must be taken into consideration as lower-limb sagittal plane kinematics (e.g. hip/knee angles) are closely coupled^4–6^.

Previous studies have used visual feedback to allow healthy participants as well as orthopaedic and neurological patients to modify specific aspects of their walking patterns^7^. Visual feedback relies on the real-time measurement (via motion capture, electromyography, force plates, etc.) of a targeted parameter and providing quantitative information beyond what is typically available to the user. It allows for an individual to self-correct abnormal features of gait^8,9^. For example, visual biofeedback of kinematic/kinetic parameters has been shown to be an effective tool for improving lower-limb mechanics in patients following total knee arthroplasty^10,11^, anterior cruciate ligament reconstruction^12^ and stroke^13–16^. Furthermore, healthy participants can use visual feedback to alter step length asymmetry^17^, foot placement^18^, and knee or hip flexion angles^19^ while walking as well as improve lower-limb mechanics during long distance running^20,21^. Still, these studies provide feedback of just one aspect of gait and focus only on endpoint or peak measurements without prioritizing temporal specificity. Because gait deficits often involve multiple abnormalities that occur at specific points in the gait cycle, it is difficult to determine which would be the most effective to target for rehabilitation. Additionally, previous studies using visual feedback were not concerned with how the manipulation of one aspect of gait impacted walking kinematics globally. To constrain these interactions, it would be necessary to provide simultaneous visual feedback of multiple aspects of walking. Delivering additional streams of visual feedback, however, comes at the cost of added attentional load^22–24^, which has been shown to hinder walking performance^25–27^. The purpose of this study is to determine how we can most effectively deliver multiple channels of kinematic information to alter a walking pattern in a temporally-specific manner.

A series of studies has shown that people can use multiple dimensions of kinematic information to control a low-dimensional external device (e.g. visual cursor, wheelchair joystick, etc)^28–32^. Here, we look to utilize similar dimensionality reduction principles to change multiple facets of a walking pattern simultaneously. We have created a novel feedback system that uses principal component analysis (PCA) to weight multiple channels of kinematic information and display the participant’s performance as a simple one-dimensional ‘summary’ of their walking pattern relative to a prescribed goal pattern. We focused on sagittal ankle position trajectories such that participants had to alter their kinematics along the AP and vertical axes within each stride in order to match a prescribed goal stride. Thus, the feedback combined four dimensions of information (i.e. two for each ankle) to produce a one-dimensional summary of walking performance.

To evaluate the utility of this novel feedback, we compared performance to participants who received four concurrent streams of one-dimensional, Cartesian-based visual feedback (i.e. one for each dimension) to learn the same goal pattern. Cartesian feedback is similar to conventional gait training in which a single stream of visual feedback contains information of one aspect of gait. We investigated the rate and extent to which healthy participants could use these two types of feedback to alter their walking pattern with the hypothesis that reducing the dimensionality of the visual feedback using principal component analysis will allow for improved performance in this skilled walking task.

## Methods

### Participants

Thirty young, healthy adults (10 per condition; PC feedback, Cartesian feedback, and PC match) were recruited for this experiment (15 men, 15 women; mean age ± SD: 23.5 ± 4.0 yr). All participants provided written, informed consent before taking part in the experiment. The experimental protocol was approved by the Johns Hopkins Medicine Institutional Review Board and all experiments were performed in accordance with relevant guidelines and regulations. All participants were free of any neurological and musculoskeletal conditions.

### Motion analysis

**Figure 1:**
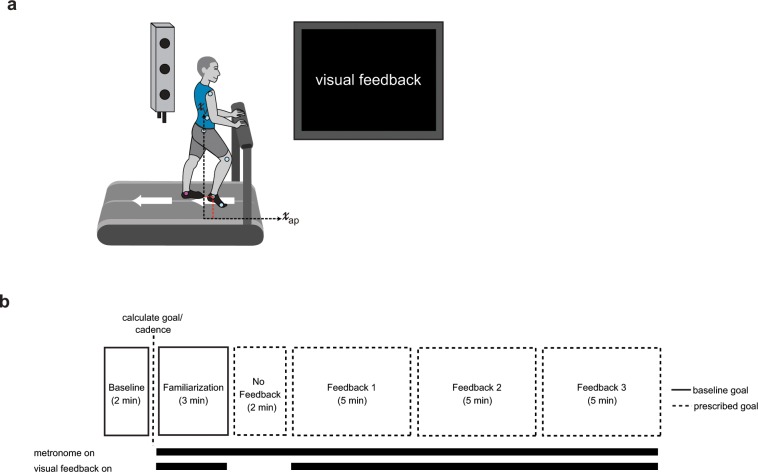
Experimental set up and paradigm visual feedback display. (**a**) Marker placement and general set up for motion capture. Left and right ankle position was recorded in the sagittal plane. (**b**) We calculated the goal walking pattern and goal cadence from the 2 minutes of baseline walking at 1 m/s. Participants were given 3 minutes of familiarization where the goal ‘target’ line corresponded to their baseline walking. During No Feedback, participants were instructed to walk naturally in rhythm with the metronome but did not receive performance feedback. In Feedback 1–3, participants were instructed to minimize the deviation from the goal target line(s) by altering their walking pattern. The goal target line(s) corresponded to the modified walking pattern. Participants were instructed to walk in rhythm with the metronome during these blocks. Each feedback block was 5 minutes.

We recorded participants’ kinematics using an Optotrak Certus motion capture system (Northern Digital, Waterloo, ON) as they walked on an instrumented treadmill (Woodway, Waukesha, WI) that allowed us to detect right and left foot contacts. The belt speed was set to 1 m/s for the entirety of the experiment. Kinematic data were collected at 100 Hz from 12 infrared-emitting diodes placed bilaterally on the foot (fifth metatarsal head), ankle (lateral malleolus), knee (lateral joint space), hip (greater trochanter), pelvis (iliac crest), and shoulder (acromion process; Fig. 1a).

### Experimental paradigm

Participants walked on a custom-built treadmill which was also controlled through Vizard. Walking speed was set to 1 m/s for all walking trials (the belts were always tied at the same speed). Participants were instructed to stand in the middle of the treadmill with one foot on each belt so that we could detect heel strikes from the force plates for visual feedback display. They wore a safety harness that was suspended from the ceiling to protect against the risk of falling. The harness did not provide any body weight support. While walking, participants were instructed to walk with their arms across their chest.

All groups experienced the same experimental paradigm that consisted of six blocks: (1) Baseline, (2) Familiarization, (3) No Feedback, (4) Feedback 1, (5) Feedback 2, and (6) Feedback 3 (Fig. 1b). The groups differed by the type of visual feedback given (i.e. PC or Cartesian, described below in ‘Visual Feedback’). During the Baseline block, participants walked naturally at 1 m/s for 2 minutes. From this baseline walking, we calculated the goal stride, variable weights, and metronome cadence as detailed below in ‘Goal Walking Pattern Calculation’ and ‘Visual Feedback’. During the Familiarization block, participants walked with the visual feedback for 3 minutes to get accustomed to the visual display and metronome as they walked. The ‘target’ line(s) were set to the participants’ baseline walking so that if they walked naturally, their visual feedback was close to the goal. Participants were informed that their performance did not matter during this block. Thus, they were free to explore the feedback and gain experience walking in beat with the metronome. During the No Feedback block participants were instructed to walk naturally in beat with the metronome. This block was designed to gather baseline performance. Next, participants experienced three identical, 5-minute Feedback blocks. During these blocks, participants responded to the visual feedback such that they had to alter their natural walking pattern toward a goal walking pattern to improve task performance. As the participants improved performance in the task, their walking kinematics progressed toward the goal walking pattern.

### Goal walking pattern calculation

**Figure 2:**
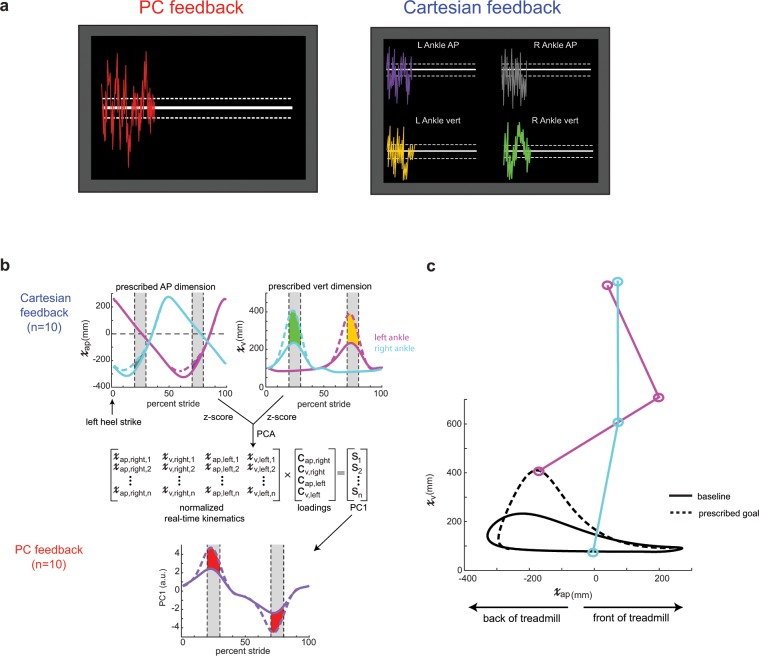
(**a**) Visual feedback display for PC feedback and Cartesian feedback. Participants were instructed to change their walking pattern so they minimized the distance from the target line(s). PC feedback had one target line while Cartesian feedback had four target lines (one for each kinematic dimension). Dashed lines around the target line correspond to the success zone. (**b**) Calculation of the prescribed goal ankle kinematics. The pink trace denotes the left ankle while the cyan trace denotes the right ankle kinematics. AP and vertical dimensions were multiplied by Gaussian gains with maximum magnitudes of 0.75 and 2.5, respectively, over swing phase. Cartesian feedback displayed the difference between goal and real-time kinematics, displayed as the colored shaded regions during mid-swing on each leg. The gray shaded regions denote where this difference was averaged for visual feedback display (i.e. the rewarded time windows). We ran principal component analysis on the goal kinematics to calculate loadings. These loadings were used to calculated a goal PC1 for the PC feedback as well as a real-time PC1 using the normalized real-time kinematics. PC feedback displayed the difference between the goal and real-time PC1, displayed as the red shaded region during mid-swing on each leg. (**c**) Goal and baseline sagittal plane ankle kinematics for the left ankle. Participants had to take shorter, higher steps to improve performance in both dimensions. The right ankle had an identical goal pattern.

The purpose of this study was to assess how to best deliver visual feedback containing information of multiple kinematic dimensions to instruct a new walking pattern. The desired walking pattern was calculated from each participant’s baseline walking (collected during the ‘Baseline’ block) such that the vertical dimension of the ankle position was increased relative to baseline and the antero-posterior (AP) dimension of the ankle position was decreased relative to baseline (Fig. 2b,c). All kinematics were computed in a hip-centered coordinate system to ensure that any whole-body translation on the treadmill did not affect the output. We focused the goal kinematics on the swing phase (i.e. subjects had to take shorter, higher steps) rather than the stance phase because stance is largely constrained by the speed of the treadmill belts. To avoid discontinuities in the kinematics at the stance-swing transitions, the goal kinematics were calculated by applying a Gaussian weighted gain over the swing phase. The gains applied to the vertical and AP dimensions were 2.5 and 0.75, respectively.

This algorithmically generated goal was used in the first condition to bias the participants’ kinematics in the direction of the goal. We refer to the participants who received this walking goal and the principal component-based visual feedback as the PC feedback group. It should be noted that the algorithmically generated goal proved to be difficult to fully reach. In a control condition, we tested how a separate set of participants performed if we gave them a more natural goal ankle trajectory instead of one that was generated algorithmically. We refer to this new set of participants as the PC match group. Participants in the PC match group received a goal pattern consisting of the average pattern of ankle kinematics that participants in the PC feedback group reached at the end of training in the original condition. The PC match group allowed us to observe if participants could use the principal component-based feedback to match an exact set of goal kinematics that we have previously observed from another group of healthy, young participants.

### Visual feedback

Participants received one of two forms of visual feedback designed to help them achieve a prescribed goal walking pattern: (1) principal component-based visual feedback that used PCA to combine four dimensions of kinematic information (Fig. 2a, left) into a single stream of performance feedback or (2) four concurrent streams of Cartesian-based visual feedback (Fig. 2a, right). Two groups (PC feedback and PC match) were tested using principal component based feedback while one group (Cartesian feedback) was tested using Cartesian-based feedback of sagittal plane ankle kinematics.

Position of the left and right ankles in the AP and vertical axes were sampled from the Optotrak software and fed into a custom Python program at real-time. Visual feedback was displayed using a Vizard development environment (WorldViz, Santa Barbara, CA) and reflected the participants’ step-by-step deviation from the desired pattern. This real-time information was displayed in a simple format, such as a trace that moves in and out of the prescribed goal zone(s) on a screen (Fig. 2a). The feedback reflected the participants’ deviation from a white ‘target’ line on the TV screen and we instructed subjects to change their pattern such that this deviation was minimized. The visual feedback was updated upon each heel strike (i.e. two new data points per stride) and tracked across the TV screen so that participants had information of their current and past performance.

**Figure 3:**
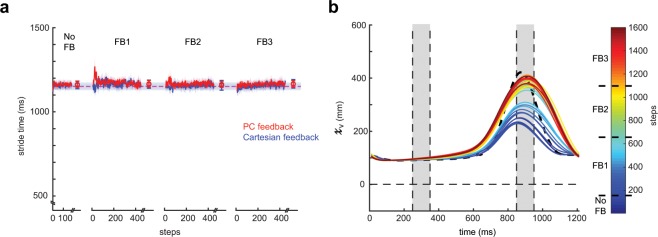
(**a**) Stride times for PC feedback (red) and Cartesian feedback (blue) across blocks. The dashed lines are the group average goal stride times provided by the metronome. Data points at the end of each block denote the group mean ± SEM of the last 50 steps within that block. All shaded regions denote SEM. (**b**) Sample participant kinematics in the vertical dimension over training. Cold colors denote baseline/early training and hot colors denote late training. Participants had to use the feedback to incrementally change their kinematics toward the prescribed goal (dashed black line) over the course of training. The vertical shaded regions denote the rewarded time windows.

For participants to use the visual feedback, it was necessary to standardize their stride time. This was because we needed to set a length of time for the goal stride from which to compare real time performance. Thus, we used a metronome to standardize the participants’ walking cadence. Participants were instructed to heel strike in rhythm with the beat from the metronome. The goal stride time was calculated from each individual’s average time between successive heel strikes during baseline walking (Fig. 3a).

The principal component-based feedback contained one stream of performance feedback around one target line (Fig. 2a, left). The position of the feedback relative to the target line in the principal component-based feedback was the difference in the first principal component (PC1) of the normalized real time stride and PC1 of the goal stride. As such, a participant who matches the goal pattern exactly (i.e. difference of zero) would receive feedback on the white target line. Weights for the real time principal component were calculated by applying PCA to the z-scored goal stride. Importantly, these weights were calculated from a given participant’s baseline kinematics collected prior to training and kept constant across the entirety of the experiment so that the participants had a constant mapping between their change in kinematics and their feedback performance.

The Cartesian-based feedback contained four streams of performance feedback, each containing information specific to a given dimension, around four target lines (Fig. 2a, right). The position of the feedback relative to each target line in the Cartesian-based feedback was determined by the difference between each of the z-scored dimensions in real time and their corresponding goal dimensions (i.e. vertical and AP ankle positions).

Participants were informed of the dimensions (AP and vertical positions of their ankles) that they could manipulate to alter their performance. For the principal component-based feedback, participants were informed that it was some combination of these dimensions that determined their feedback performance. For the Cartesian-based feedback, participants were explicitly informed which dimension corresponded to which stream of visual information. Importantly, all participants were not informed of the goal pattern; therefore, improvement in feedback performance required the participant to explore alternative walking patterns and use the visual feedback to determine which patterns resulted in better or worse performance.

For both feedback types, these differences were calculated at approximately halfway through the swing phase of each leg and then displayed at heel strike. Thus, feedback given at right leg heel strike contained information from the preceding right leg swing and left leg stance phase, and vice versa for the feedback given at left leg heel strike. This delay corresponds to approximately ¼ of the stride cycle (i.e. 250–300 ms on average). Visual feedback display and calculation are shown in Fig. 2 (panels A and B).

### Data analysis

Our measure of performance can be mapped into two spaces—PC and Cartesian space—that can be linearly transformed from one to the other using the component loadings (Fig. 2b). In each of these spaces, our primary outcome measure is the step-by-step difference between the current set of kinematics and the prescribed goal pattern. In PC space, this measure is a difference of first principal components (measured in arbitrary units) while in Cartesian space, this measure is a difference of sagittal plane ankle position (measured in millimeters). These differences are calculated and averaged over the rewarded time window (100 millisecond windows centered approximately around mid-swing of each leg; displayed as vertical dashed lines between 20–30 and 70–80 percent stride in Fig. 2b). As such a value of zero for these measures represents perfect performance in matching the prescribed goal pattern over these time windows. We use PC space as a measure of overall performance while we use Cartesian space to break out how individual dimensions evolve over the course of training. We ensured that participants were maintaining the cadence provided by the metronome by calculating stride time between consecutive left heel strikes.

Performance during the all blocks of the experiment was calculated by averaging over specific time periods in the experiment. For each of the feedback blocks, we averaged performance over the first and last 50 steps in each block to obtain a measure of early and late performance, respectively. Early performance was our measure of rate of increased performance while late performance was our measure of task proficiency at the end of each block.

To demonstrate that participants in PC match could exactly match the set of goal kinematics, we calculated the root mean squared error (RMSE) between the kinematics observed at the end of Feedback 3 and the goal and compared that to those of the PC feedback group.

### Statistical analysis

The dimensions submitted to the principal component analysis were first z-scored to ensure that the absolute magnitude of the input dimensions did not drive the loading values. We used a typical principal component analysis which consists of calculating the eigenvectors of the covariance matrix of the normalized input data. We did not rotate the input data. For visual feedback, we selected the first principal component (i.e. the eigenvector that contains that highest percentage of variance from the input data) and calculated the difference between current and goal principal components.

To identify differences between groups in their ability to match the goal kinematics during training, we performed mixed design, repeated-measures ANOVA with ‘block’ (dimension 7) and ‘group’ (dimension 2) main factors. ‘Block’ was composed of performance during late No Feedback block and early and late performance from each feedback block. To observe differences between groups during specific blocks of training, we performed post-hoc analysis on the block-group interaction. Bonferonni correction for multiple comparisons was used when necessary. In addition, we used paired sample t-tests to determine which block subjects within each group reached the outer-bound of successful task performance (i.e. dashed lines in Fig. 2a). This outer-bound was selected based on the variability observed during baseline walking in pilot testing. These analyses were performed in both PC and Cartesian space. Similar mixed-methods repeated measures ANOVA design was used when analyzing group and dimension effects on loading values between PC and Cartesian as well as root mean squared error values for PC and PC Match. For repeated-measures ANOVA, we performed Mauchly’s test of sphericity and used the Greenhouse-Geisser correction of degrees of freedom if sphericity was violated. To ensure that participants remained within 60 ms (approx. 5 percent) of the prescribed stride cadence, we performed right-tailed, one-sample t-tests on the absolute difference between participants’ baseline stride times and the metronome beat interval against a null hypothesis of mean 60. Because this task relies on exploration of alternative walking patterns to achieve task success, we considered that it might be possible that subjects did not explore beyond their natural walking pattern. Thus, we used outlier analysis to exclude participants from analysis who continued to walk naturally and did not improve their performance over the course of the three feedback blocks. This analysis eliminated one subject from each of the PC, Cartesian, and PC match groups. All analyses were performed using SPSS 25.0 (IBM, Armonk, NY) and α–level was set to 0.05.

## Results

Figure 3a displays the stride times during all blocks of the experiment. Participants in the PC and Cartesian groups were able to stay within 60 ms of this prescribed walking cadence during the early and late epochs of all blocks of the experiment (all p > 0.832). Thus, we are confident that participants were able to follow instructions and complete the task.

To improve task performance, participants had to incrementally alter their walking pattern from their natural walking (example participant data for the vertical ankle movement shown in Fig. 3b) and use the visual feedback to determine if their stride-to-stride change resulted in improved or declined performance.

**Figure 4:**
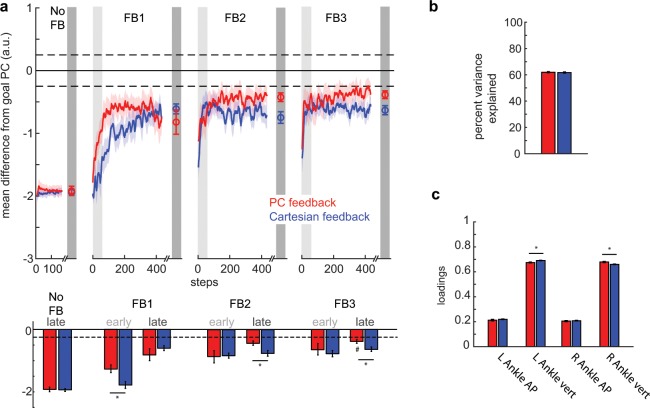
Comparison of group performance in PC space. (**a**) Display of mean difference from goal PC across blocks. Differences were calculated during the rewarded time windows for each step (i.e. mid-swing). Perfect performance is reflected by a value of 0. Dashed lines correspond to the success zone (i.e. ± 0.25 a.u. from goal). Data points at the end of each block denote the group means ± SEM of the last 50 steps for the given block. The red traces denote performance for the PC feedback group while the blue traces denote performance for the Cartesian feedback group. Colored shaded regions denote standard error. Light and dark gray shaded regions denote early and late epochs consisting of the first and last 50 steps within that block. Mean performance during these early and late epochs are displayed in the bar graphs below the time series. *Denotes a between-subject difference (p < 0.05) while # denotes a non-significant difference (p > 0.05) between performance during that epoch and the outer-bound of task success (i.e. 0.25 a.u.). (**b**) Percent variance explained by the first principal component (i.e. goal PC1) for PC feedback (red) and Cartesian feedback (blue). (**c**) Mean absolute loadings for each kinematic dimension for PC feedback (red) and Cartesian feedback (blue). These loadings were calculated from the normalized goal kinematics. *Denotes a between-subject difference (p < 0.05) and all error bars denote SEM.

Figure 4a displays our primary measure of performance (mean difference from goal PC1) for PC and Cartesian groups across all feedback blocks. Baseline performance did not vary between PC and Cartesian groups (p = 0.976; see Fig. 4a, baseline late). For analysis, we binned performance into early and late epochs (first and last 50 steps) for each feedback block. Early and late epochs, in addition to baseline measurements, were submitted to mixed-methods repeated-measures ANOVA with *block* and *group* main factors. Our analysis reveals that groups improved performance across feedback blocks (F_6, 96_ = 60.89, p < 0.001). Thus, participants were able to use the visual feedback (either PC or Cartesian) to approach the prescribed walking pattern.

Interestingly, PC and Cartesian groups’ performance varied differently across time epochs as revealed by a significant block*group interaction (F_6, 96_ = 3.23, p = 0.006). Analysis revealed that the PC group improved performance at a faster rate than the Cartesian group during Feedback 1 (p = 0.006; see Fig. 4a, FB1 early). Additionally, the PC group was closer to the goal at the end of Feedback 2 and 3 (p = 0.013 and p = 0.020, respectively; see Fig. 4a, FB2 late and FB3 late). Furthermore, performance in the PC group did not differ from −0.25 a.u. (defined as the outer-bound of task success) while the Cartesian group was not able to reach task success (t_8_ = −5.28, p < 0.001; against a null hypothesis with mean −0.25). Therefore, participants using the PC feedback demonstrate faster and more complete learning of a prescribed goal pattern during one session of training.

Information on the first principal component (PC1) for each group is summarized in Fig. 4b,c. PC1 in both groups accounted for approximately 62 percent of the variance with no difference between the groups (Fig. 4b; t_16_ = 0.35, p = 0.750). Meanwhile, group average absolute loadings for both groups were approximately 0.67 for vertical dimensions and 0.21 for AP dimensions (Fig. 4c). Mixed-methods repeated-measures ANOVA with *dimension* and *group* main factors revealed no *group* effect on loadings (F_1, 16_ = 0.68, p = 0.422). Although there was a dimension*group interaction (F_1.98, 31.66_ = 5.05, p = 0.013), which is driven by significant pairwise differences between the vertical dimensions on both the right (p = 0.006) and left (p = 0.005) sides. Still, the mean pairwise difference of the loadings in these dimensions is only 0.019 and 0.016, respectively. The statistical significance is largely driven by the small standard error within these groups (~0.005). Given that these differences in loadings only weigh the respective dimensions by 1 to 2 percent differently, we were not concerned that this difference drove our performance effect in PC space (Fig. 4a). Indeed, subsequent analysis of the kinematics reveals that these groups did differ in their walking pattern.

**Figure 5:**
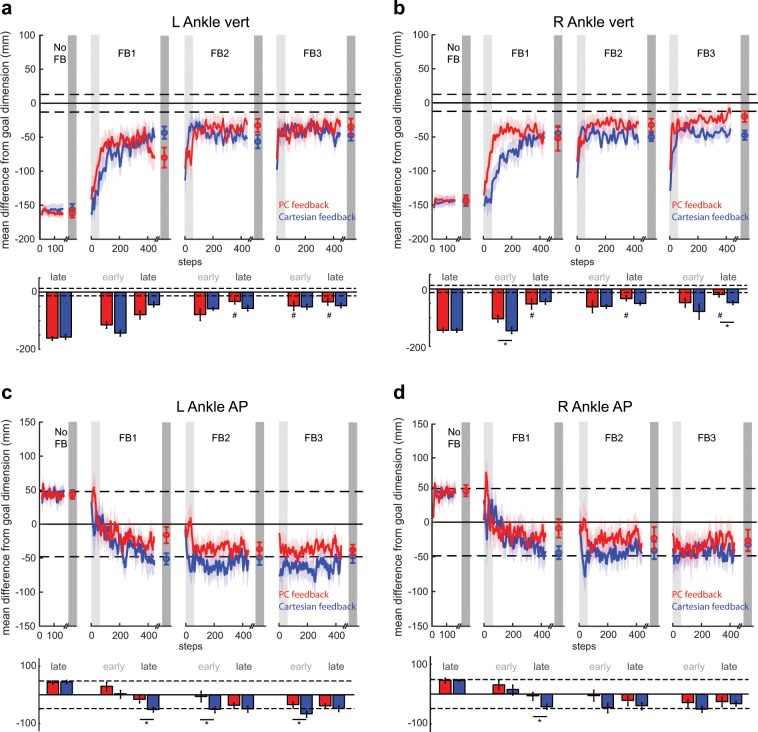
Comparison of group performance in Cartesian space. All panels show mean difference from the goal dimension for PC feedback (red traces) and Cartesian feedback (blue traces) where a value of 0 reflects perfect performance. Differences were calculated during the rewarded time windows for each step (i.e. mid-swing). Dashed lines around 0 denote the success zone for each kinematic dimension. Data points at the end of each block denote the group means ± SEM of the last 50 steps for the given block. Colored shaded regions denote standard error. Light and dark gray shaded regions denote early and late epochs consisting of the first and last 50 steps within that block, respectively. Mean performance during these early and late epochs are displayed in the bar graphs below the time series of each kinematic dimension. Data is displayed for (**a**) left ankle vertical dimension, (**b**) right ankle vertical dimension, (**c**) left ankle AP dimension and (**d**) right ankle AP dimension. *Denotes a between-subject difference (p < 0.05) for a given dimension. For the vertical dimensions, #denotes a non-significant difference (p > 0.05) between performance during a given epoch and the outer-bound of task success; determined by a paired t-test between a given participant’s outer-bound and performance during FB3 late. These are not displayed for the AP dimensions as performance is always within the success zone.

We were interested in understanding which dimensions were responsible for the difference in performance observed in PC space. Figure 5 shows the deviation of each dimension from the prescribed goal kinematics for both groups over the course of the experiment. Of note, both groups performed the same at baseline for all dimensions (all p > 0.62).

The observed difference in performance in PC space was largely due to the PC group’s ability to correct their ankle trajectory in the vertical dimension more rapidly (i.e. as measured in FB1 early) and completely (i.e. as measured in FB3 late) than the Cartesian group. Analysis revealed a significant block*group interaction for the left vertical dimension (Fig. 5a; F_6, 96_ = 3.23, p = 0.006). While analysis of the right vertical dimension did not yield a significant block*group interaction (Fig. 5b; F_2.95, 47.15_ = 1.36, p = 0.266), we did observe some epoch-specific pairwise differences between groups that contributed largely to the effects seen in PC space. Specifically, we observed a difference in performance in the right vertical dimension during both FB1 early (Fig. 5b; p = 0.022) and FB3 late (p = 0.019). These pairwise differences, along with the aggregate differences in other dimensions, are what led to the group differences observed during these epochs in PC space (Fig. 4a).

We observed that participants receiving PC feedback were able to more closely match the goal kinematics in the AP dimensions throughout the experiment (Fig. 5c,d). We observed several epoch-specific differences between groups in these dimensions. Specifically, PC showed improved performance relative to Cartesian for the left ankle AP dimension in FB1 late (p = 0.029), FB2 early (p = 0.050), and FB3 early (p = 0.049) (Fig. 5c) as well as for the right ankle AP dimension in FB1 late (p = 0.048) (Fig. 5d).

**Figure 6:**
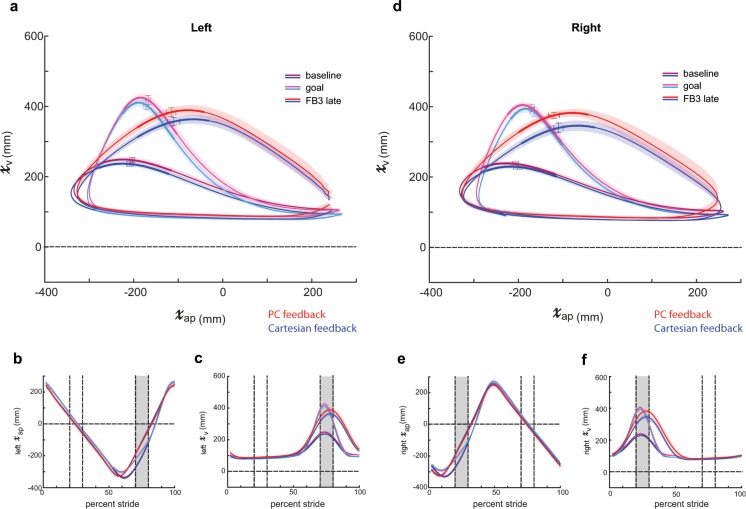
Sagittal plane ankle kinematics during late training for PC and Cartesian feedback groups (**a**) Left sagittal plane ankle kinematics for PC feedback (red shades) and Cartesian feedback (blue shades) during baseline and late training (FB3 late). Participants in both groups approach the goal kinematics (pink and cyan) by the end of training. The bolded region on each trace denotes the kinematics during the rewarded time window. Horizontal and vertical error bars within these bolded regions correspond to SEM during mid-swing in the AP and vertical dimensions, respectively. Shaded regions around each trace denote SEM in the vertical dimension. (**b**) Time-series kinematics for the left ankle AP dimension. Kinematics are aligned to left heel strike (i.e. 0 percent stride). The gray shaded region corresponds to the rewarded time window during mid-swing for this dimension. (**c**) Time-series kinematics for the left ankle AP dimension. The gray shaded region corresponds to the rewarded time window during mid-swing for this dimension. Panels (d–f) contain the same information as (**a–c)** but for the right ankle.

We then explored which dimensions of the task enabled the PC group to outperform the Cartesian group by the end of training (i.e. FB3 late). PC feedback’s level of performance at the end of feedback 3 was statistically indistinguishable from the outer-bound of task success in all four kinematic dimensions. The Cartesian group achieved task success in the AP dimensions, but did not achieve task success for the vertical dimension on both the left (p = 0.002) and right side (p = 0.001). Figure 6 displays sagittal left (panels A–C) and right (panels D–F) ankle kinematics at the end of training (FB3 late) for PC and Cartesian groups. The bolded regions of the trajectory in Fig. 6a display the portion of the left ankle trajectory within the time window (i.e. mid-swing) in which the participants receive feedback of their performance. Individual dimensions within this time window are displayed in Fig. 6b,c within the gray, shaded region between 70 and 80 percent stride. Similar information is conveyed in Fig. 6d–f for the right ankle with right mid-swing located between 20 and 30 percent stride in Fig. 6e,f. Notably, the PC group kinematics more closely match those of the prescribed goal pattern, particularly in the vertical dimensions.

Although participants in the PC group were able to more closely match the goal kinematics by the end of training, we noticed that the trajectories did not perfectly match the given goal, particularly outside of the rewarded time window (compare red traces to pink traces in Fig. 6).We considered that the original, algorithmically generated goal may have been too difficult to perform. Thus, we wanted to test if the imperfect performance in the PC feedback group was a product of the visual feedback itself or our selected goal walking pattern. Accordingly, we tested an additional group of participants—PC match—which received the identical feedback as PC but now had a goal pattern composed of the sagittal plane ankle kinematics observed in the PC group at the end of Feedback 3 (red traces in Fig. 6). In contrast to the algorithmically calculated gained up/gained down goal used previously, we know that young healthy participants have and can achieve this exact set of kinematics on the treadmill.

**Figure 7:**
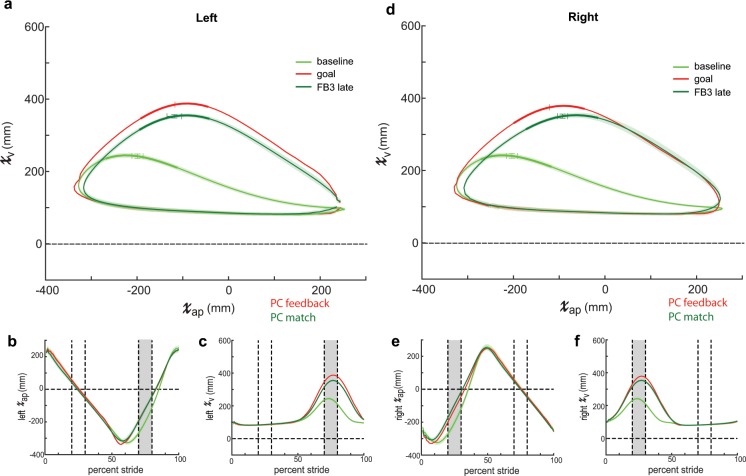
Sagittal plane ankle kinematics during late training for PC match group. (**a**) Left sagittal plane ankle kinematics for PC match (green shades) during baseline and late training (FB3 late). Participants approach the goal kinematics (red; late training performance for PC feedback group displayed in Fig. 5) by the end of training. The bolded region on each trace denotes the kinematics during the rewarded time window. Horizontal and vertical error bars within these bolded regions correspond to SEM during mid-swing in the AP and vertical dimensions, respectively. Shaded regions around each trace denote SEM in the vertical dimension. (**b**) Time-series kinematics for the left ankle AP dimension. Kinematics are aligned to left heel strike (i.e. 0 percent stride). The gray shaded region corresponds to the rewarded time window during mid-swing for this dimension. (**c**) Time-series kinematics for the left ankle AP dimension. The gray shaded region corresponds to the rewarded time window during mid-swing for this dimension. Panels (d–f) contain the same information as (**a**–**c**) but for the right ankle.

**Figure 8:**
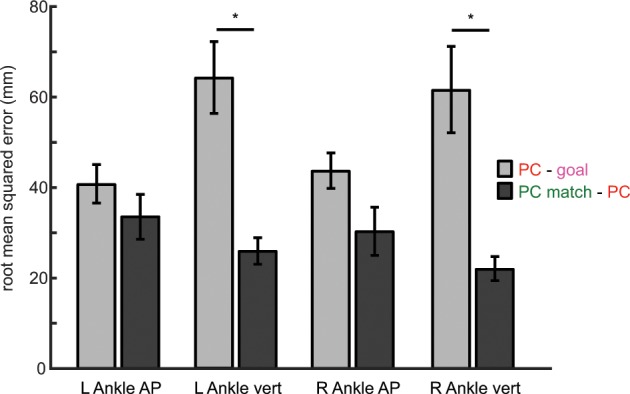
Root mean-squared error between late training and goal kinematics for PC feedback (light gray) and PC match (dark gray) groups in each kinematic dimension. An error closer to a value of 0 corresponds to the participants more closely matching the goal kinematics. Color scheme in the legend corresponds to the colors of the kinematic traces displayed in Figs. 5 and 6. Error bars denotes SEM. *Denotes a between-subject difference (p < 0.05) for a given kinematic dimension.

We found that PC Match were able to use the PC feedback to more closely match their respective goal pattern than the PC group (Fig. 7). Specifically, mixed-methods repeated-measures ANOVA revealed a significant between-subject effect of *group* (F_1, 16_ = 16.97, p = 0.001) as well as a significant group*dimension interaction (F_1.60, 25.63_ = 7.46, p = 0.005) on RMSE (Fig. 8). Post-hoc analysis revealed pairwise differences between groups’ RMSE for the left vertical dimension (p < 0.001) and right vertical dimension (p = 0.001). These results suggest that the imperfect performance observed in the PC group was due to our selection of goal kinematics. Thus, given an appropriate goal, PC feedback can be used to teach an exact set of kinematics.

## Discussion

In this study, we have shown that more information is not necessarily better when using visual feedback to achieve a modified walking pattern. The participants in this study were able to use a single stream of PC information to change multiple features of their gait toward the prescribed goal pattern and outperformed those receiving Cartesian-based information. Specifically, we found that healthy, young participants could use the principal component-based visual feedback to learn a prescribed goal pattern at a faster rate and more completely than those who received concurrent feedback of all dimensions. These results suggest that this novel principal component-based visual feedback can be used as a straightforward summary of walking performance that enables us to alter multiple aspects of gait toward a given goal pattern. Our findings demonstrate that this novel approach could be promising for rapidly and intuitively teaching persons with pathological gait how to simultaneously correct multiple gait abnormalities.

PCA is a commonly used algorithm when studying movement. For example, it has been used to characterize walking features^33–36^ or map high dimensional movement to low-dimensional control of a device (i.e. body-machine interfaces)^28–32^. Here we used PC-weighted feedback to simultaneously teach participants multiple aspects of movement toward a prescribed movement pattern. This is novel as we are not aware of any work that has previously used this approach. PC feedback offers specific advantages. Using PCA to reduce the dimensionality of visual feedback presents a solution to the high-dimensionality of learning a new walking pattern. Improved performance using this feedback requires concurrent improvement in multiple kinematic dimensions. For example, a subject that progresses toward the goal pattern in one dimension but experiences an equal and opposite decline of performance in a different, equally-weighted dimension will not receive rewarding feedback for that particular set of kinematics. Thus, PC feedback constrains unwanted changes in kinematics while rewarding a change in walking pattern toward the prescribed goal on a whole-gait level. Additionally, PC feedback allows for simplicity of feedback such that participants can respond to an intuitive, low-dimensional form of feedback. They can simultaneously receive feedback of multiple aspects of their walking pattern without having the added attentional load of multiple streams of visual information.

While both groups changed their walking pattern toward the prescribed goal pattern, the current results show participants receiving PC feedback displayed a faster and more complete change in performance than those receiving visual feedback containing four concurrent streams of kinematic information. Our primary measure of performance is the step-by-step difference from goal in PC space as this metric incorporates the aggregate performance from the individual weighted kinematic dimensions. This metric can be considered a standardized deviation from the prescribed goal pattern on a whole-gait level. We were able to see differences between PC feedback and Cartesian feedback when comparing single sessions of training. Thus, PC feedback has good potential for training a new set of walking kinematics more effectively than Cartesian feedback.

We believe that PC feedback allowed for more rapid improvement of performance for two reasons: 1) condensing the visual information to a single stream of visual information reduces the attentional demands of the task and 2) the PC feedback preferentially up-weighted the most important features of movement that required change to progress toward the walking goal. Previous studies have shown that motor performance declines with increased attentional demands^25–27^. PC feedback not only reduced the number of streams of visual information that the participants were required to attend to, but also uses a weighted sum to produce a single stream of information. Participants receiving the PC feedback are motivated (via their performance gains) to preferentially improve the dimensions that are more heavily weighted. That is, an improvement (in millimeters) of a dimension with a higher weighting produces a more significant performance gain than an equal improvement of a dimension with a lesser weighting. On the contrary, participants who received Cartesian feedback did not have access to this weighting and changes in all dimensions resulted in equal performance gains when fed back to the participant. This advantage of PC feedback is demonstrated when comparing the improvement in the vertical dimensions of the ankle trajectory. These vertical dimensions were weighted more heavily than the AP dimensions (Fig. 4c) and showed the most differential improvements across feedback types (Fig. 5a,b).

A key attribute of both feedback types in this study was temporal specificity of performance feedback. For participants to gain the most information from the visual feedback, we had to ensure that they could meaningfully assign a change in feedback performance to a change in their kinematics. For this reason, we chose to display two discrete measures of performance per stride (i.e. at left and right heel strikes) that contained information during the most informative portion of the gait cycle needed to reach the goal pattern (i.e. mid-swing). As such, the visual feedback relayed the performance of the step immediately preceding its presentation. This temporal specificity introduces an additional constraint to participants’ performance that most other gait retraining studies do not consider. A majority of studies using visual feedback to alter gait focus on scanning the entire gait cycle for either peak or endpoint kinematics^10,15,17–20,37^. While participants may be able to improve their performance using this feedback, gait deficits are often temporally specific within a given stride^38,39^. Thus, we wanted to address not only the extent to which they are adjusting their walking pattern but also constrain when they are making these adjustments.

The findings presented here also highlight the importance of selecting an appropriate goal pattern. In our first condition, we demonstrate that participants can use PC feedback to improve performance within the selected time-windows (Figs. 4a and 5). However, we observed imperfect performance in portions of the swing phase outside of the time-windows (Fig. 6). Indeed, our original intention was to create an algorithmically generated goal to bias participants in the direction of the goal kinematics, not necessarily to achieve a perfect match. However, we contend that our original, algorithmically generated goal required coordination at the hip and knee joints that was not easily attained, which hindered achievement of the exact set of ankle kinematics over the entire stride cycle. To prove that the observed limit in performance in the original PC group was due to goal selection and not a product of the feedback, we observed a second condition in which our goal kinematics were adjusted to a more natural ankle trajectory (Fig. 7). Subsequently, participants were able to use PC feedback to more closely achieve the goal over the entire gait cycle (Fig. 8). Thus, given an appropriate goal walking pattern, participants are capable of using PC feedback to match an exact set of kinematics.

A number of previous studies have used visual feedback to alter sagittal plane ankle trajectories^40–44^. The novelty of this study lies in the type of feedback used to bias the participants in the direction of the goal kinematics. We selected these kinematic features as a method for testing PC-based feedback in a healthy population. Ultimately, PC feedback can be used for any variety of kinematic features in any desired plane of movement. The only necessary ingredient is a goal template for the chosen kinematic features that differs from the current movement. Given the goal template, the feedback will algorithmically weight the most relevant features of movement and feedback the performance in an intuitive, summary format. We believe this feedback has applicability to a wide range of fields—from motor rehabilitation to athletic performance.

While this study reveals promise in PC feedback as a viable method for teaching multiple temporally-specific aspects of walking, there are some limitations. Because we did not test for participants’ ability to retain the modified walking pattern, we are hesitant to term their improved performance ‘learning’. Additionally, this study does not test for the generalization of this modified pattern to other walking patterns or other environmental contexts (e.g. overground). Many past studies have described using visual feedback to modify gait as skilled locomotor learning^41,42,44,45^. Our results and methodology compare favorably to these past studies; thus, we can best describe our task as a skilled motor task. While participants can use PC feedback to improve task performance, more studies are needed to test for the long-term retention and generalization of the modified walking pattern for us to confidently conclude PC feedback induces learning. Indeed, our goal for this study was to use the feedback to increase performance using a variety of visual feedback types and compare performance across feedback types.

We think that these results have potential implications for both healthy and pathological movement. We have created a novel visual feedback system for teaching multiple aspects of modified walking pattern in an intuitive, low-dimensional way. The current results demonstrate how we can more effectively deliver multiple streams of kinematic information for the users to change their walking patterns toward a prescribed goal. Thus, if we have a high-dimensional, coordinated movement we would like to train, PC feedback can bias users toward that movement in a low-dimensional, intuitive way. For healthy movement, this could mean training a more efficient running style, a modified golf swing, or altered free-throw shooting mechanics, among others. Further, if we consider gait disorders that contain multiple kinematic abnormalities, PC-based feedback may present a viable method for teaching a new set of kinematics toward a more ‘healthy’ gait pattern. Consider, for example, a patient with stiff-knee gait following stroke. Beyond decreased knee flexion, stiff-knee gait typically presents with multiple kinematic abnormalities such as hip circumduction, vaulting, or pelvic tilt^46,47^. PC-based feedback offers a method for not only addressing the primary deficit (i.e. decreased paretic knee flexion) but also simultaneously addressing the accompanying deficits/compensatory movement. In addition, this feedback offers a method for algorithmically weighting patient-specific features of gait. Given a set of baseline kinematics, the principal component analysis will produce a set of weights that are appropriate for a given individual, thus accommodating the heterogeneity that is present in pathological movement. Ultimately, we aim to apply these findings to the rehabilitation of those with gait disorders.

## Data Availability

The datasets analyzed during the current study are available from the corresponding author on reasonable request.
